# Open access versus subscription journals: a comparison of scientific impact

**DOI:** 10.1186/1741-7015-10-73

**Published:** 2012-07-17

**Authors:** Bo-Christer Björk, David Solomon

**Affiliations:** 1 Hanken School of Economics, Helsinki, Finland; 2 College of Human Medicine, Michigan State University, East Lansing, MI, USA

**Keywords:** impact, open access, peer review, scientific publishing

## Abstract

**Background:**

In the past few years there has been an ongoing debate as to whether the proliferation of open access (OA) publishing would damage the peer review system and put the quality of scientific journal publishing at risk. Our aim was to inform this debate by comparing the scientific impact of OA journals with subscription journals, controlling for journal age, the country of the publisher, discipline and (for OA publishers) their business model.

**Methods:**

The 2-year impact factors (the average number of citations to the articles in a journal) were used as a proxy for scientific impact. The Directory of Open Access Journals (DOAJ) was used to identify OA journals as well as their business model. Journal age and discipline were obtained from the Ulrich's periodicals directory. Comparisons were performed on the journal level as well as on the article level where the results were weighted by the number of articles published in a journal. A total of 610 OA journals were compared with 7,609 subscription journals using Web of Science citation data while an overlapping set of 1,327 OA journals were compared with 11,124 subscription journals using Scopus data.

**Results:**

Overall, average citation rates, both unweighted and weighted for the number of articles per journal, were about 30% higher for subscription journals. However, after controlling for discipline (medicine and health versus other), age of the journal (three time periods) and the location of the publisher (four largest publishing countries versus other countries) the differences largely disappeared in most subcategories except for journals that had been launched prior to 1996. OA journals that fund publishing with article processing charges (APCs) are on average cited more than other OA journals. In medicine and health, OA journals founded in the last 10 years are receiving about as many citations as subscription journals launched during the same period.

**Conclusions:**

Our results indicate that OA journals indexed in Web of Science and/or Scopus are approaching the same scientific impact and quality as subscription journals, particularly in biomedicine and for journals funded by article processing charges.

## Background

### Emergence and growth of open access

Over the last 20 years the publishing of scientific peer-reviewed journal articles has gone through a revolution triggered by the technical possibilities offered by the internet. Firstly, electronic publishing has become the dominant distribution channel for scholarly journals. Secondly, the low cost of setting up new electronic journals has enabled both scholars and publishers to experiment with new business models, where anybody with internet access can read the articles ('open access' or OA) and the required resources to operate journals are collected by other means than charging readers. Similarly, increased availability can be achieved by scientists uploading the prepublication versions of their articles published in subscription journals to OA web repositories such as PubMed Central. The majority of publishers now allow some form of archiving in their copyright agreements with authors, sometimes requiring an embargo period. Major research funders such as the National Institutes of Health (NIH) and the Wellcome Trust have started requiring OA publishing from their grantees either in open access journals (gold OA) or repositories (green OA). A recent study showed that 20.4% of articles published in 2008 were freely available on the web, in 8.5% of the cases directly in journals and in 11.9% in the form of archived copies in some type of repository [1].

In the latter half of the 1990s when journals created by individual scientists were dominating OA publishing, these journals were not considered by most academics a serious alternative to subscription publishing. There were doubts about both the sustainability of the journals and the quality of the peer review. These journals were usually not indexed in the Web of Science, and initially they lacked the prestige that academics need from publishing. Quite often their topics were related to the internet and its possibilities, as exemplified by the *Journal of Medical Internet Research*, which in 15 years has managed to become a leading journal in its field.

A second wave of OA journals consisted of established subscription journals, mainly owned by societies. These publishers decided to make the electronic version of their journal(s) freely accessible. Such journals are particularly important in certain regions of the world for example, Latin America and Japan, where portals such as Scielo and J-stage host hundreds of journals at no cost to the publishers. One of the earliest journals to make its electronic version OA was *BMJ*, which since 1998 has made its research articles freely available.

The third wave of OA journals was started by two new publishers, BioMedCentral and Public Library of Science (PLoS). They pioneered the use of article processing charges (APCs) as the central means of financing professional publishing of OA journals. Since 2000 the importance of the APC business model for funding OA publishing has grown rapidly. BioMedCentral was purchased in 2008 by Springer and over the last couple of years almost all leading subscription publishers have started full open access journals funded by APCs. The leading scientific OA journals using the APC model tend to charge between US$2,000 and US$3,000 for publishing but overall the average APC was US$900 in 2010 across all journals charging APCs listed in the Directory of Open Access Journals [2]. In many fields the payment of such charges is a substantial barrier to submissions. In a broad survey of authors who had published in scholarly journals, 39% of respondents who hadn't published in OA journals mentioned problems in funding article-processing fees as a reason [3].

Subscription publishers have also tried an OA option called hybrid journals where authors can pay fees (typically in the range of US$3,000) to have the electronic versions of their articles OA as part of what is otherwise a subscription journal. The uptake for hybrid journals in general has been very limited at about 1% to 2% for the major publishers [4].

### Does OA threaten to undermine scientific peer review?

The starting point for this study are the claims made, often by publishers and publishers' organizations, that the proliferation of OA would set in motion changes in the publishing system which would seriously undermine the current peer review system and hence the quality of scientific publishing. Suber has written an excellent overview of this discussion [5]. Lobbying using this argument has in particular been directed against government mandates for OA such as implemented by the NIH for their grantees. It is claimed that the resulting increase in posting of manuscript copies to OA repositories would lead to wide-scale cancellation of subscriptions putting traditional publishers, both commercial and society in jeopardy and in the long run result in an erosion of scientific quality control. This scenario is based on the assumption that the OA publishers would take over an increasing part of the publishing industry and would not provide the same level of rigorous peer review as traditional subscription publishers, which would result in a decline in the quality of scholarly publishing. The NIH have documented that their mandate has not in fact caused any harm to publishers [6].

The critique has in particular been focused on OA publishers that charge authors APCs. Superficially such publishers would seem to be inclined to accept substandard articles since their income is linearly dependent on the number of papers they publish. There have in fact been reports of some APC-funded OA publishers with extremely low quality standards [7]. Reports of such cases in the professional press such as the recent article 'Open access attracts swindlers and idealists' [8] in the *Finnish Medical Journal*, a journal read by the majority of practicing physicians in Finland, can by the choice of title alone contribute to a negative image of OA publishing. The founding of the Open Access Scholarly Publishers Association, which in particular strives to establish quality standards for OA journals, was in part a reaction by reputable OA publishers to the appearance of such publishers on the market.

One of the questions in the above-mentioned survey of scholarly authors [3], dealt with the 'myths' about open access, including the quality issue. On a Likert scale researchers in general tended to disagree with the statements 'Open access undermines the system of peer review' and 'Open access publishing leads to an increase in the publication of poor quality research' (results reported in Figure 4; [3]). It thus seems that a majority of scholars or at least those who completed this very widely disseminated survey did not share this negative perception of the quality of OA publishing.

### Aim of this study

Scientific quality is a difficult concept to quantify. In general terms very rigorous peer review procedures should raise the quality of journals by screening out low quality articles and improving manuscripts via the reviewers' comments. In this respect one could assume that the novel peer review procedures used by certain OA journals such as *PLoS ONE *should lower the quality. However, such journals essentially leave it to the readers to affirm the quality through metrics such as the number of citations per article. In practice the only proxy for the quality that is generally accepted and widely available across journals are citation statistics. In the choice of title for this article we have hence consciously avoided the term scientific 'quality' and chose to use 'impact' instead, which is closely related to citations such as in the impact factor used in Journal Citation Reports.

It has now been 20 years since the emergence of the first OA journals and 10 years since the launch of the first major OA journals funded by APCs. The number of peer-reviewed articles published in OA journals was already around 190,000 in 2009 and growing at the rate of 30% per annum [9]. Roughly half of the articles are published in journals charging APCs [2]. Enough time has also passed so that the qualitatively better OA journals and in particular journals that have been OA from their inception are now being indexed by major citation indexes such as the Web of Science and Scopus. In the last few years academic search engines such as Google Scholar have also emerged, but the data generated by these automated searches is too unstructured to be used for a study of the citation counts of large numbers of articles or full journals. In contrast both the Journal Citation Reports (JCR), and SCOPUS via the data available on the SCImago portal provide aggregated data in the form of impact factors, which can be used for comparing OA and subscription journals.

This provides empiric data enabling us to ask meaningful questions such as: 'How frequently are articles published in OA journals cited compared to articles in non-OA journals?'. Although the citation level cannot directly be equated to scientific quality, it is widely accepted as a proxy for quality in the academic world, and is the only practical way of getting comprehensive quantitative data concerning the impact of journals and the articles they contain. The aim of this study was thus to compare OA and subscription journals in terms of the average number of citations received both at the journal and article level.

### Earlier studies

Over the past 10 years there have been numerous studies reporting that scientific articles that are freely available on the internet are cited more frequently than articles only available to subscribers (for overviews see Swan [10] and Wagner [11]). Most of these studies have been conducted by comparing articles in subscription journals where some authors have made their articles freely available in archives. Gargouri *et al*. [12] found a clear citation advantage of the same size both for articles where the author's institution mandated OA, and for articles archived voluntary. They also found that the citation advantage was proportionally larger for highly cited articles. Some authors claim that when eliminating factors such as author's selecting their better work for OA dissemination, the advantage, at least concerning citations in Web of Science journals is low or even non-existent. Evans and Reimar using extensive Web of Science data report an overall global effect of 8% more citations, but with a clearly higher level of around 20% for developing countries [13]. Davis, in a randomized trial experiment involving 36 mainly US-based journals, found no citation effect but a positive effect on downloads [14]. His study was however limited to high-impact journals with wide subscription bases.

Assuming that there is some level of citation advantage, this would mean that the articles published in full OA journals would receive an additional citation advantage beyond their intrinsic quality from their availability. In practice it would, however, be very difficult to separate out the effects of these two underlying factors. A share of the articles in subscription journals (approximately 15%) also benefit from the increased citations due to the existence of freely available archival copies as noted for instance by Gargouri *et al*. [12]. If there was a consensus of the citation advantage for being freely available, it would be possible to correct for this effect. Since the estimates of this factor vary so much across studies, we are hesitant to attempt such a correction.

However, we don't necessarily need to explicitly take this factor into account when assessing the quality level of the global OA journal corpus. If articles in them on average get as many citations as articles in subscription journals, then their overall scientific impact (as measured by getting cited) is also equal. OA is just one of several factors influencing the citation levels of particular journals, others being the prestige of the journals, the interest of the topics of the articles, the quality of the layout for easy reading, timeliness of publication and so on.

Journals that were launched as OA from relatively new publishers such as PLoS or BMC have disadvantages in other respects. They lack the established reputation of publishers that have been in business for decades. The reputation of these journals is also hindered by a large, though shrinking, number of researchers who believe that electronic-only OA journals are somehow inferior to their more established subscription counterparts. In this study we will therefore make no attempt to look separately at the citation effect of OA, due to the complexity of the issue and the lack of a reliable estimate of the effect.

There are a few previous studies that have tried to determine the overall quality of OA journal publishing as compared to traditional subscription publishing. McVeigh studied the characteristics of the 239 OA journals included in the 2003 Journal Citation Reports [15]. Her report contains very illustrative figures showing the positions of these journals in the ranking distribution within their respective scientific disciplines. Overall, OA journals were represented more heavily among the lower-ranking journals, but there were also 14 OA journals in the top 10% in their disciplines. She also mentions that 22,095 articles were published in these OA journals in 2003. In considering the results from this early study it is important to bear in mind the highly skewed regional and age distributions of the journals in question. Only 43% of the OA journals were published in North America or Western Europe, and the vast majority of the journals were old established journals that had recently decided to make their electronic content openly available.

Giglia [16] set out to duplicate the McVeigh study, to the extent possible. Giglia was now able to rely solely on the DOAJ index for info about which journals were OA and identified 385 titles to study, using JCR from 2008 as the starting point. Giglia studied the distribution of titles in different percentiles of rank in their discipline using the same breakdown as McVeigh. All in all the results were not much different from the earlier study. Giglia found that 38% of the 355 OA journals in Science Citation Index and 54% of the 30 OA journals in Social Science Citation Index were in the top half ranks in JCR.

Miguel *et al*. [17] focused on studying how well represented gold and green OA journals were in citation indexes. They were able to combine DOAJ data with data from the SCOPUS citation database, which covers more journals than JCR, and could also use the average citation counts from the SCImago database. The results highlighted how OA journals have achieved a share of around 15% of all SCOPUS indexed journals for Asia and Africa and a remarkable 73% for Latin America. Of particular interest for this study was that some of the figures in the article showed the average number of citations per document in a 2-year window (calculated over journals) for particular journal categories. Thus the overall average number of citations was around 0.8 for OA journals, 1.6 for subscription journals allowing green posting and 0.8 for subscription journals not allowing green posting. They found highly differentiated average citation levels for nine different broad disciplines. They also found very clear differences in the citation levels between regions, with North American and European OA journals performing at a much higher level than journals from other parts of the world. Both in the disciplinary and regional breakdowns the non-OA journals followed the same patters, so that the relative performance of OA journals to non-OA journals was relatively stable.

## Methods

The data for this study were obtained from four databases. These included Ulrichsweb, Journal Citation Reports 2010 (JCR), SCImago Journal & Country Rank (SCImago), and the Directory of Open Access Journals (DOAJ). SCImago and DOAJ are openly available and provide their data in an easily downloaded format. Both our institutions have subscriptions to the electronic versions of Ulrichsweb and JCR, and it was possible to use our institutional access to these databases to obtain the information needed.

Ulrichsweb is a database of detailed information on more than 300,000 periodicals of all types. The JCR is the 2010 version of a database concerning the articles published and the citations received by the peer-reviewed journals indexed in the Web of Science citation index, a database of selected high quality scholarly journals maintained by Thomson Reuters. This study largely focuses on the average number of citations received by a journal over the most recent 2-year period, commonly called an impact factor. SCImago provides open access to similar metrics for citations concerning journals included in the Scopus Citation Database maintained by Elsevier. Scopus is similar to Web of Science but provides data on a larger number of journals. The DOAJ is a database of open access journals that provides basic information about the journals as well as immediate unrestricted access to full text articles for some of these journals. Of these services, Web of Science whose citation index is provided through the JCR has the strictest inclusion criteria, followed by Scopus. DOAJ accepts all journals that fulfill certain criteria concerning the open accessibility and the peer review, whereas Ulrichsweb is open for any journal to self-report their data.

A limitation of this method is that journals not indexed in Web of Science or Scopus cannot be included, since there is no way to obtain citation data in a systematic way. Google scholar could be used to study citations in that index to individual journals but the process is extremely labor intensive and cannot be performed for large numbers of journals.

Studies have shown a high degree of correlation between the citation metrics of JCR and Scopus, although their absolute values differ. For instance Pislyakov [18] studied the citedness of 20 leading economics journals using data from both JCR and Scopus and found that the correlation between the Impact factors of these two indexes was 0.93 (Pearson). Sicilia *et al*. [19] also found a strong correlation between the two measures for computer science journals. Hence either one provides a good measure for the level of citations.

We used this mix of sources because we needed a number of data items for our analysis that could not be obtained from just one database. Ulrichsweb was used to obtain the start year for each journal as well as the up to five discipline categories in which it was classified. It was also used to identify the country of origin of the publisher. Being listed in the DOAJ was used as an indicator of whether a journal was open access and to determine if a journal charged APCs. The JCR was used to obtain the 2-year impact factor for each journal as well as the number of articles published in it in the most recent year available in the report, 2010. SCImago was used to obtain the 2-year citation count divided by number of articles published for Scopus indexed journals (in essence similar to the JCR impact factor) and the number of articles published in 2011.

To create a merged data set for analysis we started with the Ulrichsweb database, first narrowing the database to only journals that were: abstracted or indexed, currently active, academic/scholarly, refereed, and formatted as online and/or in print.

We selected all journals within those limits that were listed in the following discipline categories (based on the discipline coding used by Ulrichsweb): arts and literature; biological science; business and economics; chemistry; earth, space and environmental sciences; education; mathematics; medicine and health; physics; social sciences; technology and engineering. While there were other disciplines categorized in Ulrichsweb, these in our view captured the major scholarly disciplines. Many journals were listed under multiple disciplines. We recorded each discipline listed for each journal. The maximum for any journal was five. The data were retrieved in January 2012.

We then merged data from the other three databases to the journals identified in Ulrichsweb using either the International Standard Serial Number (ISSN) or the Electronic International Standard Serial Number (EISSN) as the identifier. There were 23,660 journals identified in Ulrichsweb meeting the criteria within the 11 disciplines of which 12,451 (52.6%) were in the SCImago database as of January 2012, 8,256 (35.0%) were in the JCR 2010 and 2,530 (10.7%) were in the DOAJ as retrieved from their web site in August 2011.

Citation metrics of OA and subscription journals were analyzed in two different ways. Firstly they were analyzed with journals as the unit of analysis, which was at the level the data were retrieved from the four databases. We also estimated the citation metrics of the articles published. This was performed by weighting the journal level citation metrics by the number of articles published in each journal per year using article counts provided by the JCR and SCImago databases. This lends more or less weight to each journal based on the number of articles that were published within the journal. We feel this adds a new and important dimension to the analysis as compared to earlier studies.

In the data collection and analysis process we found some problems with the SCImago data. The site allows downloading the basic article numbers and citation data for all journals as one Microsoft Excel file with the most current year's data. The data on impact factors and number of articles was for 2011 but it seems that the article and citation counts are not complete for the full year, so that both the article numbers and impact factors are too low. This could easily be checked for individual journals and it turned out that the impact factors for 2010 as well as preceding years were in most cases almost double compared to the 2011 figures. A comparison with the journal level analysis in Miguel *et al*. [17] also pointed in the same direction. Unfortunately it was not possible to extract the older data for the over 12,000 journals in the study so we were limited to using the 2011 data, which was incomplete.

We nevertheless feel that the analysis using SCOPUS data provides a useful triangulation with the JCR analysis. Provided that the insufficient counting for 2011 is systematic across all journals, with no differentiation between OA and subscription journals, the citation levels for OA vs. subscription relative to each other should remain the same, although the absolute levels are lower. In comparing the numbers with the JCR based the proportions between OA and subscription citation rates were approximately the same in both sets supporting the conclusions we later illustrate mainly with the JCR results.

## Results

The results were calculated using 2-year average citations (impact factors) from the JCR and Scopus (via SCImago) by journal and weighted by the number of article in each journal as described above. OA and subscription journals were compared by the time period when they were launched (pre-1996, 1996 to 2001, and 2002 to 2011), by country published grouped into the four largest publishing countries (USA, UK, The Netherlands, and Germany) versus other countries, scientific discipline (medicine and health versus other) and business model (OA funded by APC, OA not funded by APC, and subscription).

Table 1 provides a comparison of the impact factors for OA and subscription journals based on journals in the JCR and Scopus databases. OA journals had impact factors that were approximately 76% and 67% as high as subscription journals in JCR and Scopus respectively when analyzed by journal and 73% and 62% when weighted for articles published. Due to our concerns about the Scopus data from the SCImago Journal and Country site outlined above in the Methods Section only JCR figures are presented and discussed below.

**Table 1:** The 2-year citation averages for open access versus subscription journals, calculated using Web of Science or Scopus data

Source	Type	Journals (n)	Journal level		Article level	
			
			Mean	SD	Mean	SD
Journal Citation Reports 2-year citation average	Subscription	7,609	1.97	2.95	2.81	3.31
	Open access	610	1.50	4.02	2.04	2.28
Scopus 2-year citation average	Subscription	11,124	0.85	1.38	1.59	1.99
	Open access	1,327	0.61	1.85	1.03	1.44

In the journal level calculations each journal has equal weight; in the article level calculations the impact factors for the journals are weighted by their publication volumes.

**Figure 1:**
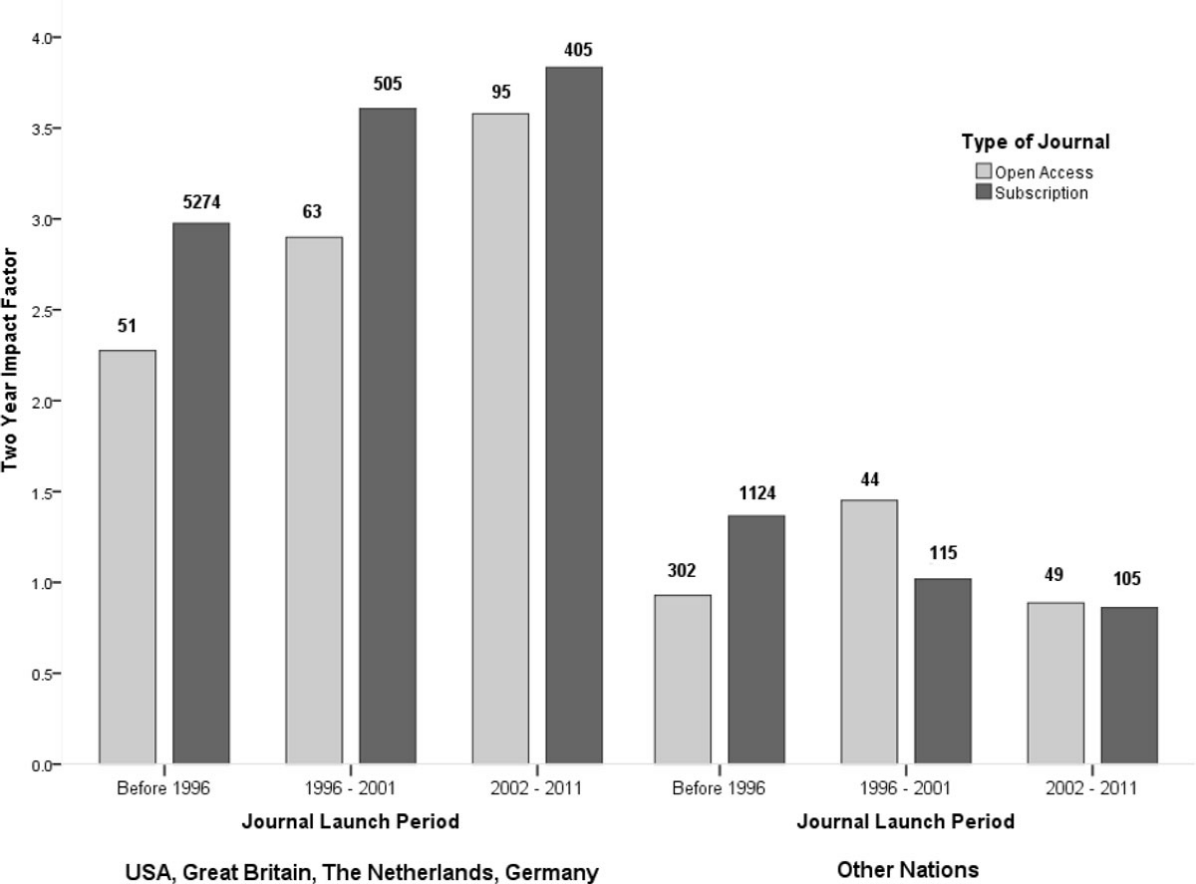
**Citation averages as a function of the journal start year for two regions**. The figures are based on Web of Science and weighted by journal article volumes.

Figure 1 shows the average JCR impact factor for OA and subscription journals weighted by the number of articles as a function of the time period the journal was launched and location of the publisher. The left side of the figure includes the journals from the four countries where most of the major society and commercial publishers are located. The publishers in these four countries account for approximately 70% of the journals in our sample. The right side of the figure includes journals publishing in the rest of the world.

There are large differences in the impact factors between the two regions with the 'big four' on average having journals with significantly higher impact factors. Somewhat surprisingly in this region more recently launched journals tended to have higher impact scores than the older more established journals. This was true for both subscription and OA journals. In addition the difference in impact between OA and subscription journals narrows with time.

The pattern for journals from the rest of the world is quite different. While the overall number of journals published is much lower, the number of OA journals is actually quite high in the pre-1996 group where OA journals have a clearly lower impact. This group largely consists of old established print journals, which at some staged have opened up their electronic versions. In the middle time period, OA journals were outperforming subscription journals and in the youngest group they were on a par with subscription journals.

### Effects of the discipline of the journals

**Figure 2:**
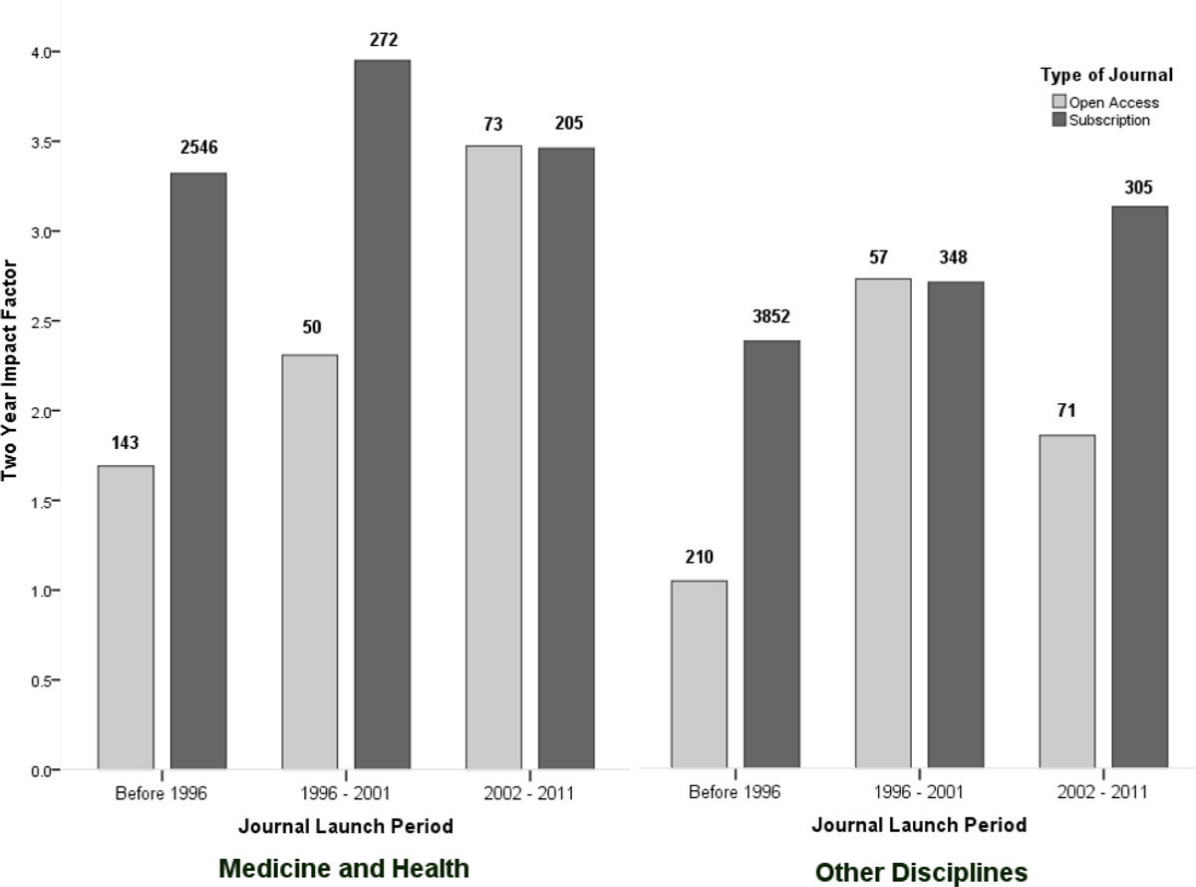
**Citation averages as a function of the journal start year for Medicine and Health versus all other disciplines**. The figures are based on Web of Science and weighted by journal article volumes.

Several studies have shown that gold open access journals have had a larger uptake in the biomedical fields [1,15], where authors usually have less problems in financing APCs and where many research funders also require some form of OA for the results. Figure 2 shows the average JCR impact factor of OA and subscription journals weighted by the number of articles as a function of the discipline. The journals were split into two groups. The first included journals with the Ulrichsweb discipline category 'Medicine and Health'. All the other disciplines were combined into the second group.

In medicine and health, the large difference in impact between OA and subscription journals seen in older journals essentially disappears among the journals launched after 2001. This probably reflects the emergence of high quality professional OA publishers such as PLoS and BioMedCentral that rely on APCs for funding. For the other disciplines, OA articles had considerably lower impact scores in journals before 1996 and journals launched after 2001 but the average impact of OA articles in journals launched between 1996 and 2001 was essentially equal to the average impact of articles in subscription journals launched in the same period. In reviewing the raw data, the high average impact of the OA articles during this period was due to a handful of relatively high impact and high volume OA journals published by BioMedCentral, which had been classified as biological rather than medical journals.

### Effects of the revenue model of OA journals

**Figure 3:**
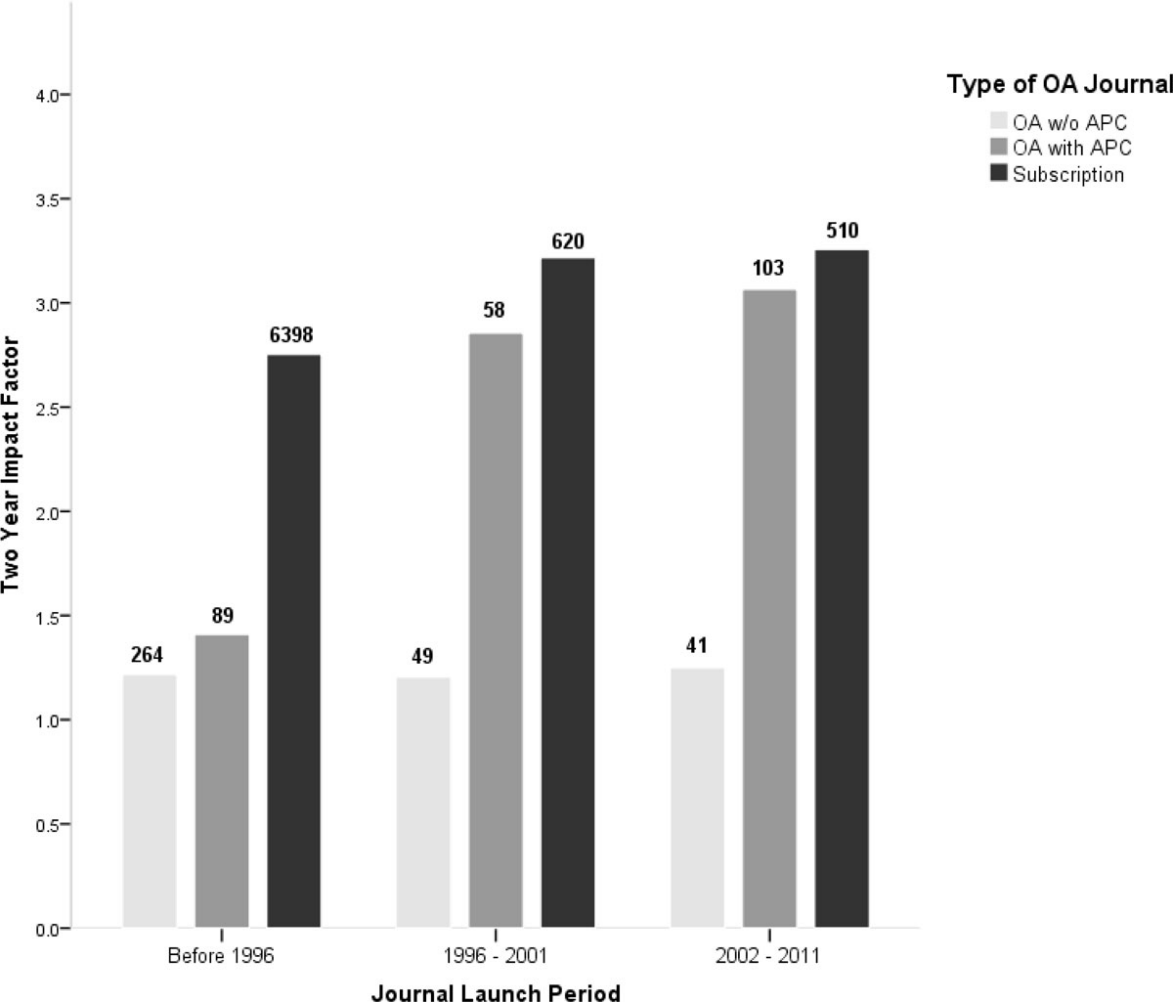
**Citation averages for open access journals using article processing charges (APCs) versus those that are free to publish in for authors, compared to impact factors for subscription journals**.

In Figure 3 (subscription journals), OA journals funded by APCs and OA journals that do not charge APCs are compared as a function of journal age. As noted above, the early OA journals were funded through volunteer effort and small subsidies from largely universities. Beginning with BioMedCentral and PLoS in about 2001 a growing number of professional publisher have begun publishing OA journals funding their operations by charging publication fees.

The impact of OA journals that are not funded by APCs are more or less the same irrespective of journal age at about 1.25. The oldest age category consists mainly of print journals that have made their electronic versions freely available. APC funded OA journals' average impact increased markedly in the period 1996 to 2001 and to a lesser extent in 2002 to 2011 nearly reaching the same level as subscription journals at about 3.2. The 89 APC funded journals launched before 1996 we expect largely include subscription journals that converted to the APC model of OA publishing. A number of the journals are published by Hindawi, which did in fact transition from a subscription publisher to an OA publisher funded by APCs [20]. The other journals are published by a variety of publishers, universities, societies and other organizations from around the world.

## Discussion

The distribution of OA journals over time periods and regions differs markedly from the corresponding distribution of subscription journals. OA journals are much more numerous in categories that have low overall impact factors which may explain some of the difference in average impact between OA and subscription journals. Almost half (302) of all OA journals found in JCR are journals started before 1996 and published in the 'other countries' region. While over 75% of the subscription journals found in the JCR were also launched before 1996, nearly 70% of subscription journals are from publishers in the four major publishing counties. As can be seen in Figure 1, across all age categories and for both OA and subscription journals, those published outside the four major publishing countries have substantially lower impact factors. While correlation is not necessary causation, the location of the publisher appears to account for much of the difference in average impact between OA and subscription journals.

The vast majority of journals founded before 1996 that are listed in the JCR started as paper-based subscription journals. Those listed as OA must at some stage have made their electronic versions open access. Many of these are journals published by scientific societies and universities but at least in one case (Hindawi) a publisher converted their whole portfolio from subscription to OA.

Both in the leading publishing countries and in the rest of the world, older established journals that have made their electronic versions openly available have lower impact scores than their subscription counterparts. This is understandable since the large commercial publishers and the leading society publishers have usually refrained from opening up the e-content, BMJ being a notable exception. But for the newer journals, particularly in medicine and health, our results show that OA journals are performing at about the same level as subscription journals, in fact getting more citations in some subcategories.

For almost 15 years the quality of OA journals has been debated and questioned. In the early days of electronic journals, when hardly any startup OA journals were operated by reputable professional publishers, it was easy to understand the reluctance of scientists to submit their best manuscripts to OA journals and for research funders and university promotion and tenure committees to accept publishing in OA journals as on par with publishing in traditional subscription based journals. After the launch of professionally run high quality biomedical OA journals beginning in about 2000, the situation has changed. Today the funding mechanism of a journal is irrelevant in considering its quality. There are large numbers of both subscription and OA journals that are high quality and widely cited.

The development and increasing acceptance of the APC funding model for OA scholarly journals has spawned a group of publishers with questionable peer review practices that seem focused on making short-term profits by having low or non-existent quality standards. Unfortunately this has created some bad publicity for OA publishing. As this study demonstrates, this does not change the broad picture. Gold OA publishing has increased at a rate of 30% per year over the past decade [9] and in the last couple of years many major subscription publishers have started adding pure OA journals to their portfolios.

We believe our study of the quality of the OA journals indexed in either Web of Science or Scopus is the most comprehensive to date. The results indicate that the level of citations for older subscription based OA journals, which have made the electronic version openly available, is clearly lower than for the corresponding subscription journals. At the same time newly founded full OA journals compete on almost equal terms with subscription journals founded in the same period. OA articles published medicine and health by publishers in the four largest publishing countries; attract equal numbers of citations compared to subscription journals in these fields. Based on the evidence from earlier studies it is likely that a part of the citations to the OA articles are due to the increased readership following from the open availability, but there is no way we can isolate the effect of this factor in our calculations nor would this factor alone account for the increasing respect researchers are showing for these journals through their citations.

The focus of the criticism of OA journals has been directed against journals funding their operations with APCs, claiming that this revenue model leads to journals lowering their review standards in order to maximize their profits. While there is clearly a substrata of journals reflecting this phenomena, there are also a growing number of high quality APC funded journals from reputable publishers that are on par with their subscription counterparts.

## Conclusions

In summary, gold OA publishing is rapidly increasing its share of the overall volume of peer-reviewed journal publishing, and there is no reason for authors not to choose to publish in OA journals just because of the 'OA' label, as long as they carefully check the quality standards of the journal they consider.

## Competing interests

There are no competing financial interests. Both authors have founded OA journals in the 1990s and are emeritus editors-in-chiefs. B-CB is a current and DS a former board member of the Open Access Scholarly Publishers Association.

## Authors' contributions

B-CB initiated the study and has written most of the background sections of the articles. DS collected the data from the different sources and made the calculations. Both authors participated equally in the analysis of the results and the drawing of conclusions.

## Authors' information

B-CB is professor of Information Systems Science at the Hanken School of Economics, Helsinki, Finland. DS is Professor of Medicine at the College of Human Medicine, Michigan State University, USA.

## Pre-publication history

The pre-publication history for this paper can be accessed here:

http://www.biomedcentral.com/1741-7015/10/73/prepub
